# Deep learning-driven multi-view multi-task image quality assessment method for chest CT image

**DOI:** 10.1186/s12938-023-01183-y

**Published:** 2023-12-06

**Authors:** Jialin Su, Meifang Li, Yongping Lin, Liu Xiong, Caixing Yuan, Zhimin Zhou, Kunlong Yan

**Affiliations:** 1 School of Optoelectronic and Communication Engineering, Xiamen University of Technology, Xiamen, 361024 China; 2 Department of Medical Imaging, Affiliated Hospital of Putian University, Putian, 351100 China; 3 School of Clinical Medicine, Fujian Medical University, Fuzhou, 350122 China

**Keywords:** Image quality assessment, Deep learning, Multi-view, Multi-task, Chest computed tomography images

## Abstract

**Background:**

Chest computed tomography (CT) image quality impacts radiologists’ diagnoses. Pre-diagnostic image quality assessment is essential but labor-intensive and may have human limitations (fatigue, perceptual biases, and cognitive biases). This study aims to develop and validate a deep learning (DL)-driven multi-view multi-task image quality assessment (M$^2$IQA) method for assessing the quality of chest CT images in patients, to determine if they are suitable for assessing the patient’s physical condition.

**Methods:**

This retrospective study utilizes and analyzes chest CT images from 327 patients. Among them, 1613 images from 286 patients are used for model training and validation, while the remaining 41 patients are reserved as an additional test set for conducting ablation studies, comparative studies, and observer studies. The M$^2$IQA method is driven by DL technology and employs a multi-view fusion strategy, which incorporates three scanning planes (coronal, axial, and sagittal). It assesses image quality for multiple tasks, including inspiration evaluation, position evaluation, radiation protection evaluation, and artifact evaluation. Four algorithms (pixel threshold, neural statistics, region measurement, and distance measurement) have been proposed, each tailored for specific evaluation tasks, with the aim of optimizing the evaluation performance of the M$^2$IQA method.

**Results:**

In the additional test set, the M$^2$IQA method achieved 87% precision, 93% sensitivity, 69% specificity, and a 0.90 F1-score. Extensive ablation and comparative studies have demonstrated the effectiveness of the proposed algorithms and the generalization performance of the proposed method across various assessment tasks.

**Conclusion:**

This study develops and validates a DL-driven M$^2$IQA method, complemented by four proposed algorithms. It holds great promise in automating the assessment of chest CT image quality. The performance of this method, as well as the effectiveness of the four algorithms, is demonstrated on an additional test set.

## Introduction

Computed tomography (CT) is commonly performed in the diagnostic radiographic examination. The image quality of chest CT affects the diagnostic decision of radiologists [1, 2], which mainly reflects in chest CT images with poor quality will make the lesion site indistinct, thus, image quality assessment (IQA) for chest CT images is very important.

CT scan will cause a certain amount of radiation to the patient; high-dose radiation is detrimental to health [3]. Radiologists may require patients with poor CT image quality to undergo additional scans or even re-scans, which leads to an increase in the amount of radiation the patient is exposed to. In most cases, CT image quality is influenced by many factors (inspiration [4], the field of view (FOV) [5], and position [6], etc.). The patient’s respiratory pattern during the CT examination can affect the quality of the CT image [4]. Incorrect respiratory pattern, leading to insufficient inspiration, is also the major fault affecting image quality [7]. Thus standard breathing instructions were used across examinations to avoid that impact: on inspiration, “take a deep breath in and hold”; on expiration, “breathe out and hold” (end-expiratory) [8]. Radiologists instruct the patient to maintain a certain posture and follow standard breathing instructions during the CT examination, the reason is that body shaking, breathing, or swallowing during the scan will lead to artifacts in CT images, which will reduce the quality and diagnosability of the images, and may even cause problems such as misdiagnosis or incomplete scan.

**Fig. 1:**
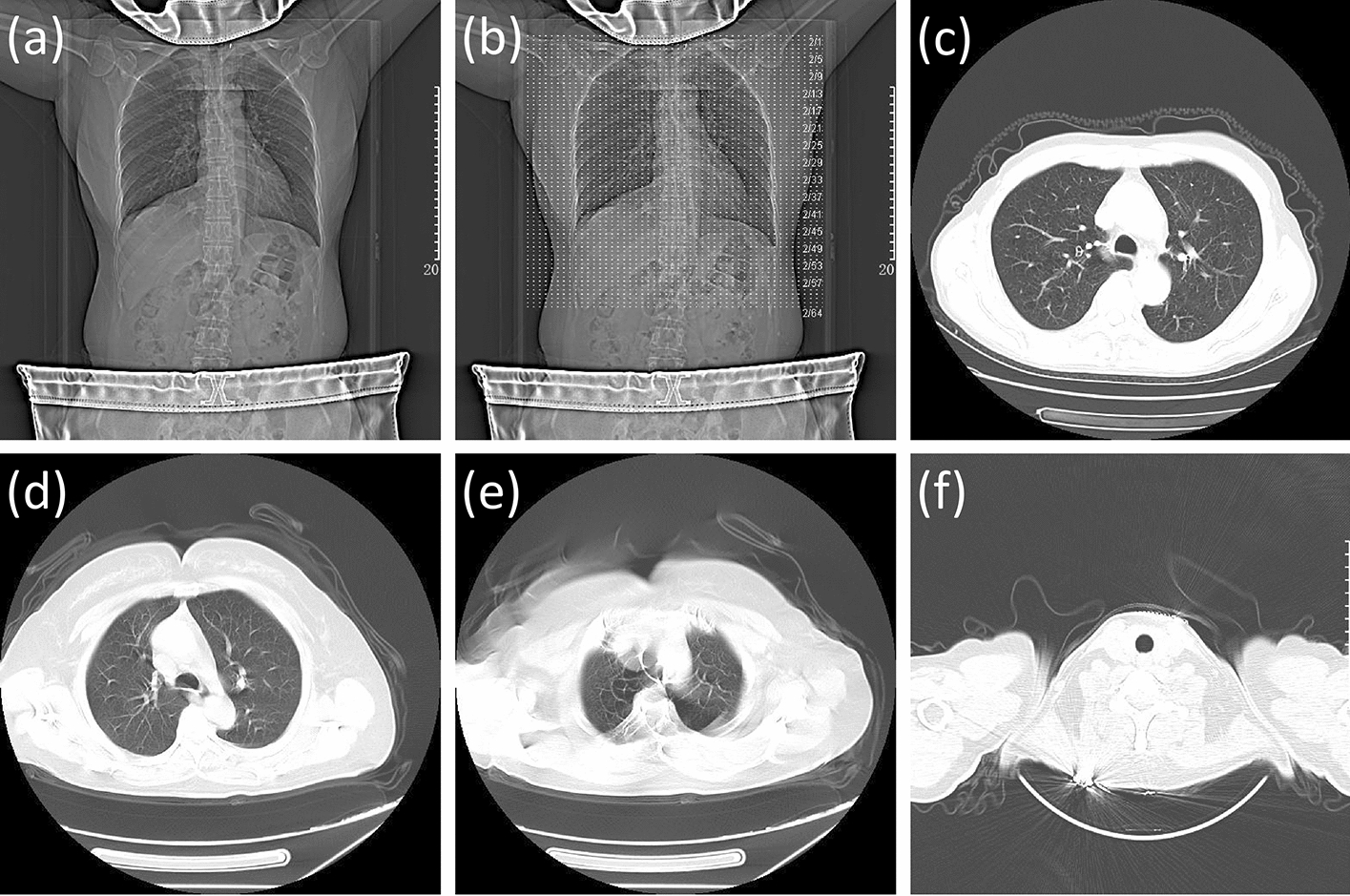
The results of patients who met and did not meet the requirements of filming. **a** Indicates that the patient raising arms above head, wearing radiation-protective products on neck and abdomen, and no metal objects while filming. **b** Is the CT image of **a** after overlaying the scan baseline. **c** Represents the patient filming at the prescribed scan FOV range, while **d** represents the patient with a portion of body outside the prescribed scan FOV range. **e** Indicates the presence of motion artifacts. **f** Indicates the presence of metal artifacts

**Fig. 2:**
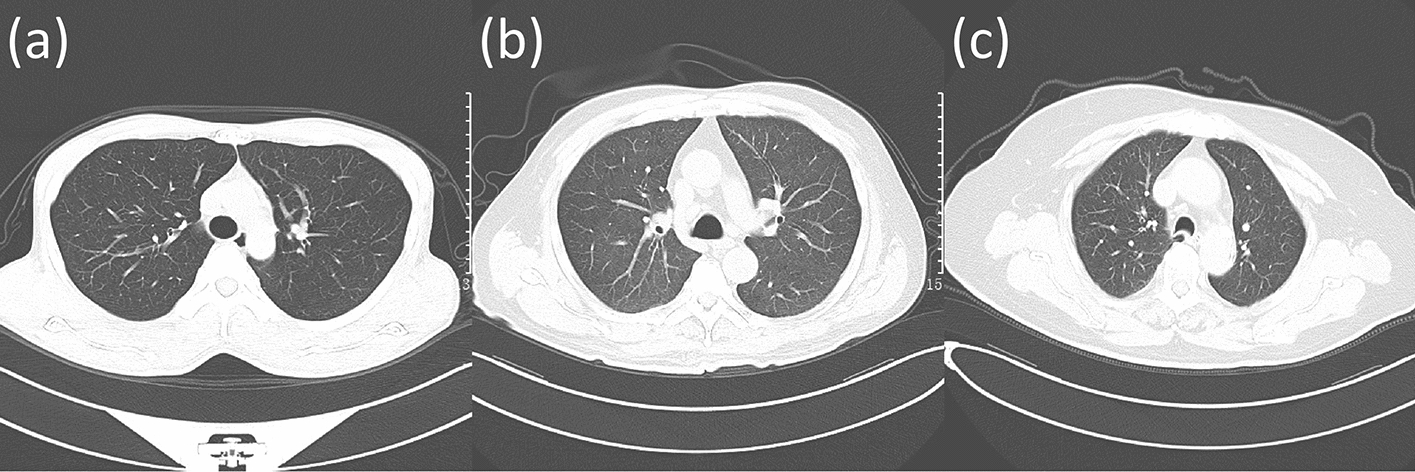
Different morphologies of tracheal carina under different levels of inspiratory adequacy. **a** Represents a sufficient inspiratory adequacy, **b** represents a average inspiratory adequacy, and **c** represents a poor inspiratory adequacy

The artifact in the CT image is one of the factors affecting the image quality, and caused not only by the inappropriate movement (i.e., respiratory motion and incorrect positioning) of the patient but also by other external factors [9]. Specifically, inappropriate patient positioning may cause motion artifacts as shown in Fig. 1e and metallic objects may cause metal artifacts as shown in Fig. 1f, both of these artifacts can decrease image quality [6, 9], thus, patients are instructed to position their arms above their head (Fig. 1a) to minimize even avoid motion artifacts [10], and take off metal jewelry before CT examination to avoid metal artifacts as much as possible, and wear radiation-protective products on their neck and abdomen to minimize radiation damage [11]. It is worth mentioning that respiratory movement can be avoided using standard breathing instructions. Whether the patient follows these instructions can be judged by observing the presence of respiratory movement-induced artifacts. In addition, comprehensive consideration of three aspects, tracheal carina morphology, bronchial beam clarity, and ribs clarity can evaluate the respiratory adequacy of the patient, and further judge the adherence to standard breathing instructions. Tracheal morphology and bronchial beam clarity will change as the patient breathes [8, 12], and the position of the ribs moves with respiratory motion [13, 14]. Specifically, based on the retrotracheal membrane configuration, patient with sufficient inspiration tended to have an ovoid tracheal carina (Fig. 2a), those with average inspiratory adequacy tended to have a bullet-shaped tracheal carina (as shown in Fig. 2b), and those with poor inspiratory adequacy tended to have a lunate shaped tracheal carina (Fig. 2c). In this study, we defined these three morphologies as convex, flat, and concave, respectively, based on the position of the retrotracheal membrane relative to the endotracheal lumen. The more sufficient inspiration, the clearer bronchial beam. There are many other factors affecting CT image quality, and the field of view (FOV) is one of them [5]. The scan FOV is determined by the X-ray source and the detector array which rotate along the central axis of the scan FOV. The CT image quality will decrease caused by truncation artifacts, which is due to the incorrect body position of the patient [15]. To avoid the generation of truncation artifacts, patients are demanded to undergo CT examination within the specified scanning FOV. Figure 1c shows the patient’s body within the prescribed scan FOV range, and Fig. 1d shows the patient’s body offset from the prescribed scan FOV. In addition, the overlayer of the Digital Imaging and Communications in Medicine (DICOM) file can be used to determine whether the region the patient receives a scan in the scanning FOV is the region of interest (ROI), which is also helpful to evaluate the image quality [16–19]. The overlayer representation is shown in Fig. 1b.

IQA is an effective approach to assess the quality of perceived visual stimuli and fall into two categories: subjective assessment by human and objective assessment by algorithms designed to mimic the subjective judgment [20]. Subjective assessment always regarded as the gold standard to evaluate images, however, IQA remains a manual evaluation process, and is limited by poor inter-observer agreement [21]. These limitations make it difficult for radiologists to make an objective diagnosis for patients undergoing CT examination, and cause energy and time waste. According to the availability of reference images, objective medical image IQA methods can be divided into three categories: (i) full reference-IQA (FR-IQA) where there is a perfect reference image for comparison with the test image; (ii) reduced reference-IQA (RR-IQA), which contains partial information of the reference image, and (iii) no reference-IQA (NR-IQA), where there is not perfect image as reference for the test image [22]. Both FR-IQA and RR-IQA are usually used for natural image evaluation, due to the availability of reference images. However, for medical image evaluation, there is no perfect medical image as reference for IQA. Therefore, in CT imaging, NR-IQA is the most appropriate method for the quality assessment of CT images.

In recent years, deep learning (DL) algorithms have been more and more widely used in many fields [23–25]. In the field of medical image processing, it provides substantial improvements for diagnosis as a Computer Aided Diagnosis (CAD) tool. The image processing applications of magnetic resonance image (MRI), CT image, ultrasound image and other radiological images usually include classification [26], segmentation [27], and detection [28]. Deep neural networks (DNNs), as a promising option to solve the NR-IQA task, can automatically extract deep features related to image quality assessment and optimize these features through backpropagation methods to improve prediction performance [29]. In 2019, Kashyap et al. [30] proposed an automatic classification method based on DenseNet121 architecture, to detect suboptimal anterior–posterior (AP) chest radiographs caused by technical deficiencies such as over- or under-exposure or wrong positioning of the patients. Although the automatic classification of AP chest radiographs with or without technical deficiencies achieved a specificity of 100% and an area under the receiver operator curve (AUC) of 0.93, they did not target identifying the specific reason for failure along with the determination of the need for repeat radiograph. In 2021, Nousiainen et al. [31] used a variety of ResNet50 and DenseNet121 networks, to estimate the lung inclusion, patient rotation, and inspiration on posterior–anterior (PA) chest radiographs. Although the model performed well on two test datasets, the scoring ambiguity (inter-observer variability) raises some bias in model performance. Poggenborg et al. [32] developed a real-time Artificial Intelligence (AI) image quality feedback tool, to help radiologists analyze whether PA chest radiographs were adherence to desired standards of collimation, patient rotation and inspiration or not right after the completion of the examination at the X-ray system. Compared to the image quality prior to the use of the real-time AI image quality feedback tool, there was indeed a relative increase of images with optimal image quality with respect to collimation, patient rotation and inspiration, which was achieved by 30%. However, the tool only evaluated image quality in three aspects, and there was only a relative increase of 4% of images with optimal inspiration. In 2022, Meng et al. [33] develop a fully automatic system to assess the image layout and position of chest radiographs, which used an encoder–decoder network that was similar to the U-Net framework to perform landmark detection and image segmentation. Although the system provided assessments similar to the mean opinion scores (MOS) of radiologists regarding image layout and position, and the mean absolute perception error (MAPE) of the layout was 3.05%, and that of the position was 5.72%, inspiration, the important factor affecting image quality, was not under consideration.

As a summary, most studies have primarily addressed IQA in chest radiographs, with limited exploration in the realm of chest CT image. In addition, there are many factors that affect image quality, and there are even fewer studies on chest CT image IQA comprehensively considering multiple factors. Thus, this study presents a multi-view multi-task image quality assessment method for chest CT image IQA. The proposed method detects and segments the regions of interest (ROIs) on coronal, axial, and sagittal chest CT images, and the proposed four algorithms (pixel threshold, neural statistics, region measurement, and distance measurement) are used to mimic the reviewing strategy of radiologists.

The major contributions of this study are summarized as follows:

1. A multi-view multi-task image quality assessment (M$^2$IQA) method is presented, for chest CT image quality assessment. Compared with the previous IQA methods, the proposed method can evaluate the image quality from four aspects (inspiration, position, radiation protection, artifact), effectively screen out the chest CT images that cannot be used for the patient’s physical condition assessment, and improve prognostic accuracy and reliability.
2. Two optimization algorithms (pixel threshold and neural statistics) are proposed to enhance the accuracy of the inspiration evaluation model. The pixel threshold algorithm is utilized for assessing the tracheal carina, while the neural statistics algorithm is employed for the evaluation of bronchial beams and ribs. The effectiveness of these two algorithms is demonstrated through ablation studies.
3. Two decision algorithms (region measurement and distance measurement), are proposed to further categorize the model’s evaluation results in the position evaluation. The region measurement algorithm is utilized to determine whether the body is positioned at the center of the scan FOV, while the distance measurement algorithm is employed to assess the accuracy of aligning the start and end of the scan baseline. For the position evaluation task, the absence of these two decision algorithms would prevent the model from obtaining normal classification results, highlighting the significance of these two decision algorithms. The performance results of the position evaluation model reflect the effectiveness of these two algorithms.

## Results

In this section, the performance of the object detection model (YOLOv8) and the semantic segmentation model (U-Net) used in this study is evaluated. Additionally, ablation studies are conducted to validate the reliability of the proposed M$^2$IQA method, and a detailed discussion will be presented.

### Ablation studies

The results of the ablation studies are presented in Table 1, which are crucial for understanding the performance of different algorithms in the proposed method. It is important to note that these experiments were conducted on the additional test set to verify the robustness of the proposed method. The experimental results demonstrate that for the inspiration evaluation task, incorporating the pixel threshold (PT) algorithm or the neural statistics (NS) algorithm can improve the model’s performance in terms of precision, sensitivity, and F1-score. The combination of the PT algorithm and the NS algorithm achieves the best overall performance, with an F1-score of 0.43, which is an improvement over the baseline model by 0.11, indicating that the combined effect of the two algorithms enhances the model’s performance. The impact of the NS algorithm on the baseline model is significant, with an F1-score that is 0.11 higher than the model with the PT algorithm alone.

The experimental results validate our hypothesis that the PT algorithm can address the issue of similar tracheal carina morphology, and the NS algorithm can effectively mimic the assessment paradigm of radiologists.

Detailed statistical analysis and discussions regarding these findings will be described in section Statistical analysis and discussion of ablation results.

**Table 1:** The ablation result of four different models

	Precision	Sensitivity	Specificity	F1-score	*P*-value
Baseline	0.20	0.80	0.56	0.32	1.09E−07
Baseline + PT	0.20	0.80	0.56	0.32	1.20E−07
Baseline + NS	0.33	0.60	0.83	0.43	8.44E−01
Baseline + PT+NS (M$^2$IQA)	0.33	0.60	0.83	0.43	8.43E−01

### Comparative studies

Due to the relatively simple ROI segmentation requirements in this study, the commonly used U-Net as a semantic segmentation model is sufficient to meet the segmentation needs of the method. However, some ROIs in this experiment, such as bronchial beams and ribs, are difficult to detect. Therefore, multiple object detection models were trained and their performances were compared. It is important to note that, to ensure accuracy, only bounding boxes with an intersection over union (IoU) greater than 0.70 were considered. Table 2 presents the experimental results of testing multiple object detection models on the test dataset. The results indicate that YOLOv8 outperformed other models (YOLOv7, RetinaNet, CenterNet, Faster R-CNN) in all three metrics (precision, sensitivity, F1-score). Specifically, YOLOv8 achieved the highest F1-score in each evaluation sub-parts, demonstrating superior performance. Therefore, YOLOv8, which exhibits good generalization ability, was selected as the target detection model for the M$^2$IQA method.

Table 3 represents the training time of the DL models.

Detailed statistical analysis and discussions regarding these findings will be described in section Statistical analysis and discussion of observer study results.

**Table 2:** The comparison results of the five evaluation sub-parts under five different detection models

Sub-parts	Evaluation metrics	YOLOv8 (avg ± std)	YOLOv7 (avg ± std)	RetinaNet (avg ± std)	CenterNet (avg ± std)	Faster R-CNN (avg ± std)
Artifact	Precision	1.00 ± 0.00	1.00 ± 0.00	0.99 ± 0.00	0.00 ± 0.00	0.95 ± 0.01
	Sensitivity	1.00 ± 0.00	1.00 ± 0.00	0.98 ± 0.02	0.00 ± 0.00	1.00 ± 0.00
	F1-score	1.00 ± 0.00	1.00 ± 0.00	0.99 ± 0.01	0.00 ± 0.00	0.97 ± 0.01
Arms position	Precision	0.99 ± 0.00	0.96 ± 0.00	0.96 ± 0.00	0.00 ± 0.00	0.88 ± 0.00
	Sensitivity	0.96 ± 0.00	0.91 ± 0.00	0.89 ± 0.00	0.00 ± 0.00	0.91 ± 0.00
	F1-score	0.97 ± 0.00	0.94 ± 0.00	0.92 ± 0.00	0.00 ± 0.00	0.90 ± 0.00
Radiation protection	Precision	0.99 ± 0.02	0.99 ± 0.02	1.00 ± 0.00	0.50 ± 0.71	0.88 ± 0.04
	Sensitivity	0.97 ± 0.01	0.90 ± 0.04	0.52 ± 0.52	0.01 ± 0.02	0.95 ± 0.03
	F1-score	0.98 ± 0.00	0.94 ± 0.01	0.60 ± 0.47	0.02 ± 0.03	0.92 ± 0.03
Rib	Precision	0.90 ± 0.08	0.77 ± 0.01	0.79 ± 0.13	0.25 ± 0.50	0.16 ± 0.19
	Sensitivity	0.82 ± 0.11	0.71 ± 0.15	0.74 ± 0.14	0.01 ± 0.02	0.14 ± 0.18
	F1-score	0.85 ± 0.06	0.73 ± 0.01	0.76 ± 0.13	0.02 ± 0.03	0.14 ± 0.17
Bronchial beam	Precision	0.90 ± 0.09	0.63 ± 0.10	0.86 ± 0.07	0.60 ± 0.43	0.37 ± 0.04
	Sensitivity	0.92 ± 0.01	0.62 ± 0.09	0.88 ± 0.03	0.36 ± 0.25	0.44 ± 0.15
	F1-score	0.91 ± 0.05	0.62 ± 0.10	0.87 ± 0.03	0.44 ± 0.29	0.40 ± 0.09

**Table 3:** The training time of the DL models

	Tracheal carina	Bronchial beam	Rib	Arms position	Scan baseline position	Body position	Radiation protection	Artifact	Total
Model training time(h)	0.80	0.47	0.47	0.56	1.60	1.11	0.87	1.62	7.50

### Observer studies

In the observer study, the proposed method was compared with the assessment ability of radiologists on the additional test set and two public datasets (LungCT-Diagnosis [34] and CMB-LCA [35]). Four experienced radiologists, who were blinded for the study, participated in the observer study. To obtain more reliable scoring results, the MOS of the four radiologists was compared with the proposed method.In addition, three control groups were formed, consisting of M$^2$IQA vs. Ground Truth (GT), MOS vs. GT, and M$^2$IQA vs. MOS.

**Fig. 3:**
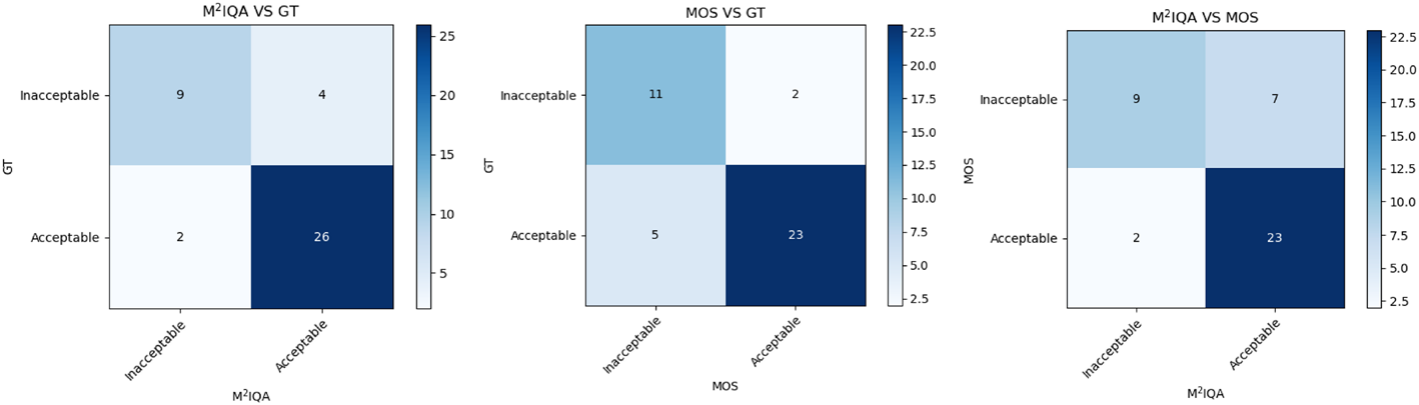
Three confusion matrices that compare the similarities and differences in evaluation results between our M$^2$IQA method, MOS, and GT on the additional test set

When evaluating on the additional test set, three confusion matrices, as shown in Fig. 3, were used to visually observe the similarities and differences in evaluation results. Based on the three confusion matrices, multiple evaluation metrics were derived and presented in Table 4.

According to Table 4, our proposed M$^2$IQA method achieved an F1-score of 0.90, while the MOS of the four radiologists’ F1-score reached 0.84, with a slight difference of 0.06 lower than our proposed M$^2$IQA method. The experimental results indicate that for the additional test set, our M$^2$IQA method’s evaluation capability is slightly superior to that of radiologists. Furthermore, the F1-score of 0.87 for the M$^2$IQA vs. MOS comparison group suggests that our proposed M$^2$IQA method demonstrates good agreement with the radiologists’ MOS.

For LungCT-Diagnosis and CMB-LCA, the evaluation focused on five specific sub-parts (artifact, tracheal carina, rib, bronchial beam, and body position) when comparing the assessment ability of the proposed M$^2$IQA method with MOS on public datasets. This selection was dictated by the absence of CT images for the evaluation of three sub-parts (arms position, scan baseline, and radiation protection). The results of multiple evaluation metrics in the three control groups on the additional test set, LungCT-Diagnosis, and CMB-LCA are presented in Table 5 when considering only these five evaluation sub-parts.

It is noteworthy that both manual and computer evaluations are time-consuming. Therefore, comparing the time consumed by the M$^2$IQA method with that of MOS on the dataset holds significance. Table 6 provides the evaluation time required for the M$^2$IQA method and MOS on three datasets.

Detailed statistical analysis and discussions regarding these findings will be described in section Statistical analysis and discussions of observer study results.

**Table 4:** The multiple evaluation metrics results of the three control groups

	Precision	Sensitivity	Specificity	F1-score	*P*-value
M$^2$IQA VS GT	0.87	0.93	0.69	0.90	0.44
MOS VS GT	0.77	0.92	0.56	0.84	0.39
M$^2$IQA VS MOS	0.92	0.82	0.85	0.87	0.18

**Table 5:** The multiple evaluation metrics results of the three control groups on three datasets

Dataset		Precision	Sensitivity	F1-score	*P*-value
Additional test set	M$^2$IQA VS GT	0.91	0.81	0.85	0.61
	MOS VS GT	0.88	0.85	0.86	0.42
	M$^2$IQA VS MOS	0.94	0.86	0.90	0.27
LungCT-Diagnosis	M$^2$IQA VS GT	1.00	0.83	0.91	0.73
	MOS VS GT	1.00	0.67	0.80	0.54
	M$^2$IQA VS MOS	0.70	0.88	0.78	0.85
CMB-LCA	M$^2$IQA VS GT	0.86	0.75	0.80	0.45
	MOS VS GT	0.86	0.75	0.80	0.36
	M$^2$IQA VS MOS	0.86	0.86	0.86	0.90

**Table 6:** The evaluation time of the M$^2$IQA method and the MOS on different datasets

		Dataset					
		Additional test set		LungCT-Diagnosis		CMB-LCA	
		M$^2$IQA	MOS	M$^2$IQA	MOS	M$^2$IQA	MOS
	Tracheal carina	0.00 ± 0.00	4.19 ± 1.24	0.00 ± 0.00	5.97 ± 1.61	0.00 ± 0.00	6.16 ± 1.16
	Bronchial beam	14.67 ± 1.78	30.21 ± 3.68	3.77 ± 0.91	10.45 ± 2.21	5.21 ± 1.68	8.84 ± 2.60
	Rib	6.75 ± 0.95	25.95 ± 4.00	1.79 ± 0.41	21.02 ± 5.78	2.52 ± 0.87	17.15 ± 2.68
	Arms position	0.04 ± 0.03	0.93 ± 0.24	N/A	N/A	N/A	N/A
Evaluation time (s) (avg ± std)	Scan baseline position	0.14 ± 0.15	1.36 ± 0.40	N/A	N/A	N/A	N/A
	Body position	0.01 ± 0.00	3.64 ± 0.93	0.01 ± 0.00	4.36 ± 1.09	0.01 ± 0.00	5.05 ± 0.97
	Radiation protection	0.03 ± 0.03	1.01 ± 0.31	N/A	N/A	N/A	N/A
	Artifact	1.59 ± 0.78	6.08 ± 2.95	2.10 ± 1.11	6.91 ± 2.77	2.74 ± 1.19	7.57 ± 3.93
	Total	23.02 ± 2.86	70.07 ± 9.07	7.67 ± 2.15	48.72 ± 9.44	10.48 ± 3.29	44.78 ± 6.39

## Statistical analysis and discussion

This section will conduct statistical analysis and discussion on the experimental results mentioned above. All statistical analyzes were performed on excel (version 11.1., KINGSOFT) and p-values were obtained by two-tailed t-test, and p < 0.05 was considered a significant difference.

### Statistical analysis and discussion of ablation results

**Fig. 4:**
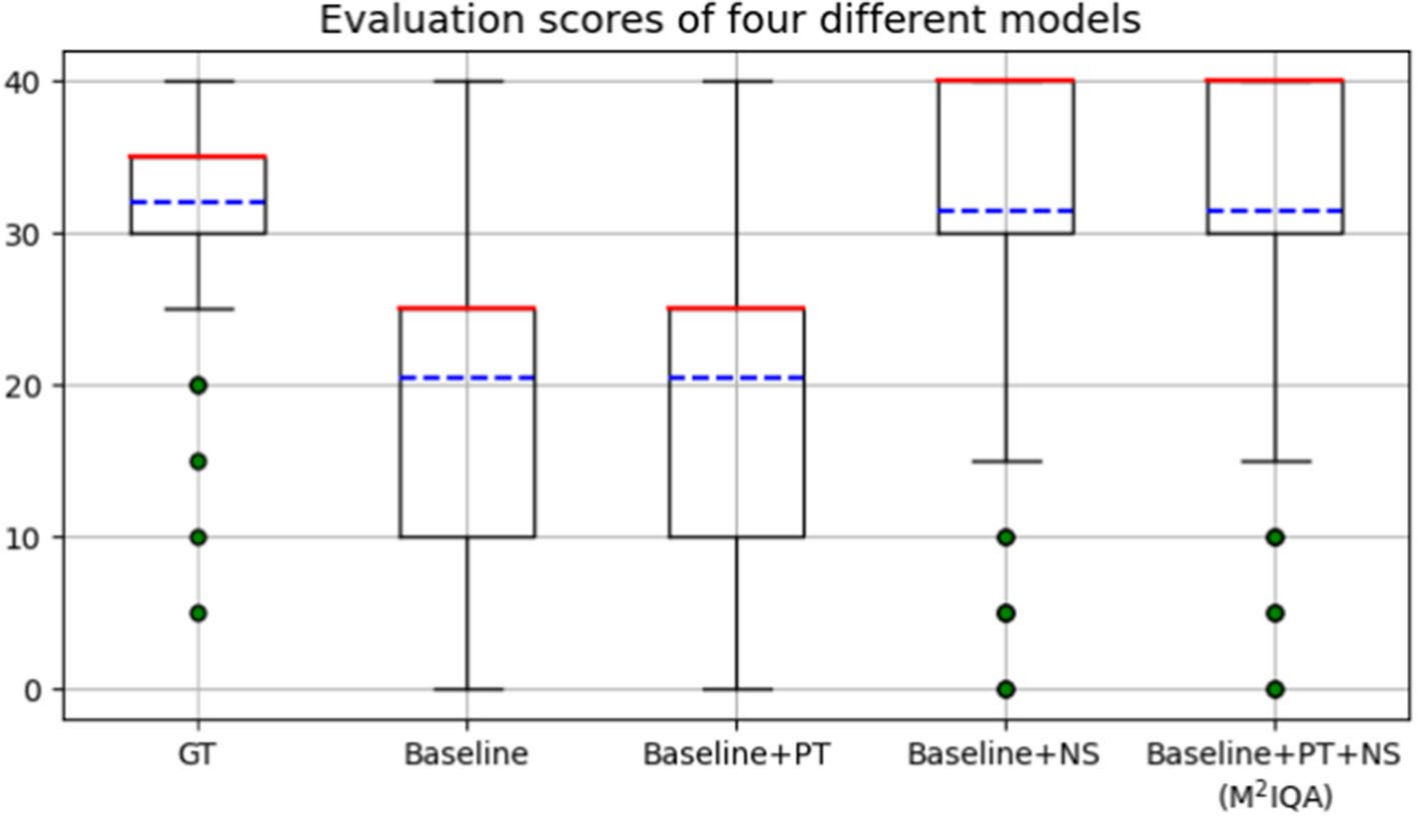
Evaluation scores of four different models

The results of the ablation studies showed that the inclusion of PT and NS algorithms improved the performance of the model. However, upon closer analysis, it was observed that the addition of the PT algorithm did not significantly improve the model’s performance in terms of precision, sensitivity, specificity, and F1-score. To better understand the impact of the PT algorithm on the model’s performance, a two-tailed t-test was performed on the evaluation scores obtained by each model, and the p-values are presented in the last column of Table 1. Additionally, the box plots shown in Fig. 4 also illustrate the differences in evaluation scores obtained by different models.

From Table 1, it can be observed that the p-value for the baseline model is 1.09E−07, indicating a significant difference between the evaluation scores of the baseline model and those of the additional test set. After incorporating the PT algorithm (Baseline+PT), the p-value increased to 1.20E−07, indicating an increased but still significant difference. The improvement of the Baseline+PT model’s evaluation score accuracy can be attributed to this reduced difference. On the other hand, the inclusion of the NS algorithm resulted in a significant improvement in the model’s performance compared to the models without NS (Baseline and Baseline+PT). This improvement could be attributed to two reasons. Firstly, the NS algorithm operates on two sub-projects (bronchial beam and rib) rather than just one (tracheal carina) like the PT algorithm. This provides additional opportunities for performance improvement. Secondly, the NS algorithm aims to imitate the evaluation paradigm of radiologists by considering multiple CT images to obtain evaluation results. This helps mitigate the impact of model accuracy because without the NS algorithm, the model derives the evaluation result from only one CT image. If the model happens to make an incorrect prediction for that particular image, it may lead to an erroneous evaluation. The NS algorithm, by considering multiple images, provides more robust evaluation results.

### Statistical analysis and discussion of comparative study results

From Table 2, it is evident that the YOLOv8 object detection model demonstrates optimal performance, thus, this study ultimately adopts it as the chosen object detection model. However, a notable observation is the notably poor performance of the CenterNet model, particularly evident in the artifact evaluation and arms position evaluation tasks. Additionally, the results across the remaining three sub-parts (radiation protection evaluation, rib evaluation, and bronchial beam evaluation) are also unsatisfactory.

The reasons for such outcomes are likely twofold. On the one hand, it is plausible that CenterNet’s inherent performance may not align well with the evaluation tasks in this study. On the other hand, it could be attributed to the study’s specific methodology aimed at achieving more accurate detection results. This methodology involves considering only bounding boxes with confidence scores exceeding 0.70, which inherently imposes higher precision requirements on each model’s detections. Under these intensified competitive conditions, CenterNet might have struggled, resulting in the appearance of zero values in precision, sensitivity, and F1-score metrics.

After conducting comparative experimental evaluations, the final model training time for each evaluation task is presented in Table 3. Due to the implementation of transfer learning, the training time for each model is relatively short, with a total training time of only 7.5 h for all eight models.

### Statistical analysis and discussion of observer study results

From Table 4, M$^2$IQA can be observed that achieve a precision of 0.87, sensitivity of 0.93, specificity of 0.69, and F1-score of 0.90, while MOS achieves a precision of 0.77, sensitivity of 0.92, specificity of 0.56, and F1-score of 0.84. All four metrics of M$^2$IQA outperform those of MOS. Specifically, precision indicates the accuracy of correctly predicting positive samples among all predicted positive samples, sensitivity reflects the accuracy of correctly predicting positive samples among all actual positive samples, specificity represents the accuracy of correctly predicting negative samples among all actual negative samples, and F1-score is the weighted harmonic mean of the first two, where higher values are desirable. The F1-score of radiologists is slightly lower than our proposed M$^2$IQA by a margin of 0.06.

The three confusion matrices shown in Fig. 3 illustrate the agreement between M$^2$IQA, MOS, and GT. From the figures, it can be observed that M$^2$IQA has 26 true positive (TP), 9 true negative (TN), and 35 correctly predicted samples, with 4 false positive (FP) and 2 false negative (FN), resulting in 6 misclassified samples. On the other hand, MOS has 23 TP, 11 TN, and 34 correctly predicted samples, with 2 FP and 5 FN, resulting in 7 misclassified samples. In the additional test set, M$^2$IQA exhibits better predictive capabilities than MOS, with a p-value of 0.44 compared to MOS’s p-value of 0.39. This suggests that both M$^2$IQA and MOS show no significant differences from GT, but the larger p-value indicates potentially smaller significant differences, implying that M$^2$IQA has better agreement than MOS.

The above experimental results indicate that when considering all evaluation tasks (eight evaluation tasks), for the additional test set, our M$^2$IQA’s assessment capability is slightly superior to that of radiologists. Furthermore, the F1-score of M$^2$IQA vs. MOS reaches 0.87, indicating a high degree of agreement between our proposed M$^2$IQA and radiologists. This to some extent indicates that our model’s assessment capability might not be inferior to radiologists’ for the data beyond the scope of our study.

The above experimental results indicate that when considering all evaluation tasks (eight evaluation tasks), for the additional test set, our M$^2$IQA’s assessment capability is slightly superior to that of radiologists. Furthermore, the F1-score of M$^2$IQA vs. MOS reaches 0.87, indicating a high degree of agreement between our proposed M$^2$IQA and radiologists. This to some extent indicates that our model’s assessment capability might not be inferior to radiologists’ for the data beyond the scope of our study.

The LungCT-Diagnosis and CMB-LCA public datasets were used to validate the above hypothesis. Due to limitations in the public datasets (lacking images required for the arms position, scan baseline, and radiation protection evaluation tasks), this experiment assessed only the remaining five evaluation tasks. The experimental results, as shown in Table 5, reveal an F1-score of 0.91 for M$^2$IQA on the LungCT-Diagnosis dataset. The MOS, with a slight difference of 0.11, is slightly lower than our proposed M$^2$IQA method. However, for the CMB-LCA dataset, M$^2$IQA achieves an F1-score of 0.80 which is the same as MOS. The p-values of M$^2$IQA vs. MOS are 0.85 and 0.90 on the LungCT-Diagnosis and CMB-LCA datasets, respectively. The results indicate that their performance does not significantly differ on different datasets, providing support for our hypothesis that our model’s assessment capability might not be inferior to radiologists’ for the data beyond the scope of our study.

The features for distinguishing different descriptive indicators are extremely subtle, and M$^2$IQA’s advantage lies in its efficient automated evaluation and assessment capabilities trained through extensive data. Human assessment (i.e., MOS) requires experienced radiologists to meticulously search, which is time-consuming and prone to fatigue-induced judgment errors. This might explain why MOS’s assessment capability lags behind M$^2$IQA. However, both M$^2$IQA and MOS have their own strengths and weaknesses. While M$^2$IQA offers stability in performance and less evaluation time (as shown in Table 6), it may struggle to recognize features it has not been trained on. On the other hand, MOS, despite underperforming M$^2$IQA in the additional test set (due to human limitations such as fatigue, perceptual biases, and cognitive biases), might provide more accurate judgments for novel cases due to its rich experience. Hence, M$^2$IQA as a computer-aided tool in collaboration with radiologists could combine their strengths and complement each other effectively.

## Conclusions and future work

In this study, a comprehensive M$^2$IQA framework for evaluating image quality in chest CT scans is proposed. Our approach combines advanced deep learning techniques with multi-view to address the challenges posed by various evaluation tasks. Through incorporating multiple scan planes and the leveraging of task-specific features, our M$^2$IQA framework effectively assesses various aspects of image quality.

Table 1 demonstrates that our proposed algorithms (pixel threshold and neural statistics) improve the model’s evaluation performance for specific tasks (inspiration evaluation). However, in the position evaluation task, the absence of our proposed algorithms (region measurement and distance measurement) renders the evaluation infeasible. It is noteworthy that the multi-view fusion strategy significantly enhances the task specificity and robustness of the model’s evaluations.

While our M$^2$IQA framework has demonstrated promising results, certain limitations remain. Future research will concentrate on refining our proposed methodology. Firstly, our dataset is not sufficiently diverse. Expanding the dataset to include a broader range of patient populations could enhance the model’s generalization capability. Secondly, the datasets used in this study are limited. Integrating our M$^2$IQA method into clinical workflows to aid radiologists in real-time image quality assessment could not only improve diagnostic accuracy and efficiency but also validate the reliability and clinical efficacy of our approach. Furthermore, investigating methods to enhance the interpretability of model predictions, such as generating heatmaps to visualize ROIs, could offer radiologists more insights for clinical decision-making. Additionally, this study focused only on four aspects of chest CT image quality assessment: inspiration evaluation, position evaluation, radiation protection evaluation, and artifact evaluation. Other factors influencing CT image quality may not have been considered, warranting further exploration in this direction.

In conclusion, the M$^2$IQA method presents a promising tool for automated chest CT image quality assessment, showcasing superior performance compared to human radiologists in the additional test set. However, further efforts are required, including dataset expansion, method integration into clinical workflows, enhanced interpretability, and exploration of additional factors influencing CT image quality.

## Materials and methods

### Datasets

The Institutional Review Board (IRB) of Fujian Putian Hospital in China approved our retrospective study, and the requirement for informed consent was waived.

The method was evaluated on a dataset consisting of 1613 images from 286 patients. The images were collected by radiologists at the Fujian Putian Hospital between January 1, 2020, and November 31, 2022. The patients were primarily scanned using two CT scanners: the SIEMENS SOMATOM DEFINITION DUAL SOURCE and the GE LIGHTSPEED. The tube voltage ranged from 120 to 150 kV, and the tube current was controlled using automatic tube current modulation technology, typically ranging from 50 to 800mA. The CT images were acquired with a thin layer thickness for the lung window, ranging from 0.625 to 1 mm.

To ensure the quality and diversity of the data used for deep learning tasks, radiologists carefully selected CT images from different scanning planes and considering the desired evaluation criteria. The dataset was divided into two parts. One part was split into three subsets with a ratio of 7:2:1 for training, validation, and testing, respectively, with the testing subset also used for comparative studies. The other part was reserved as an additional test set for the ablation studies and observer studies. It’s worth noting that a portion of both the LungCT-Diagnosis and CMB-LCA public datasets were incorporated into the observer studies, with the aim of validating the robustness and effectiveness of the proposed M$^2$IQA method across different patient populations. Table 7 provides a detailed description of the dataset composition. In the table, the dataset used for training, validation, and testing is described in terms of the number of images. On the other hand, the dataset used for the observer study is described in terms of the number of patients. This distinction is made because the two parts of the dataset serve different purposes.

**Table 7:** The detailed description of the dataset composition

Type	Training set	Validation set	Testing set	Additional test set	LungCT-Diagnosis	CMB-LCA
Inspiration				41	12	10
Tracheal carina	94	27	13			
Bronchial beam	280	80	40			
Rib	112	32	16			
Position						
Body position	335	95	48			
Scan baseline	70	20	10			
Arms position	125	35	18			
Radiation protection	32	9	5			
Artifact	82	23	12			

### Methodology

In this section, the proposed method for M$^2$IQA is introduced step by step. Firstly, the overall assessment process of the M$^2$IQA method is briefly outlined. Subsequently, detailed assessments are discussed, including inspiration, position, radiation protection, and artifact. Lastly, the multi-view fusion strategy of the M$^2$IQA method and the detailed multi-task strategy, designed to mimic the assessment paradigm of radiologists, are presented.

#### Overview of M$^2$IQA

**Fig. 5:**
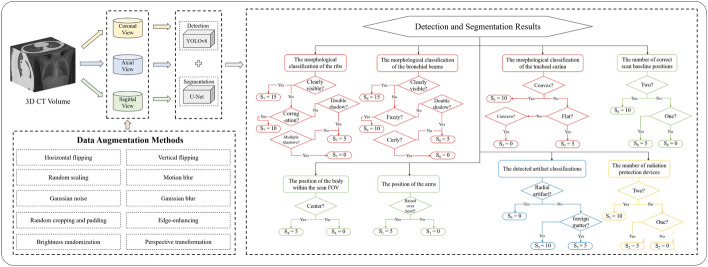
The overall evaluation flowchart of the M$^2$IQA method. The three red boxes on the right side of the image together form the inspiration evaluation task, the three green boxes together form the position evaluation task, the blue box is for the artifact evaluation task, and the yellow box is for the radiation protection evaluation task

To overcome the limitations of a single-view model, three scanning planes (coronal, axial, and sagittal) are used to comprehensively evaluate whether CT examinations produce high-quality images that are suitable for prognosis. As depicted in Fig. 5, a meticulously designed M$^2$IQA method is proposed, which considers four aspects to evaluate the image quality of patient CT images: inspiration, position, radiation protection, and artifact. Specifically, the inspiration aspect aims to assess patient inspiration adequacy from three perspectives: tracheal carina morphology, bronchial beam clarity, and rib clarity. The position aspect evaluates the patient’s position during the scan from three aspects: accurate alignment of the scan baseline at the beginning and end, the proper position of the body within the scanning FOV, and whether the arms are raised above the head. The radiation protection aspect determines the patient’s radiation protection status by examining whether the patient wears radiation-protective products on the neck and abdomen during the scan. The artifact aspect aims to identify whether the patient removed metallic objects or other objects that may interfere with the prognosis.

All the assessment results are obtained using the object detection model and semantic segmentation model. The object detection model aims to accurately enclose the ROIs with bounding boxes, while the semantic segmentation model aims to accurately segment the ROIs. By leveraging these two deep learning models, a multi-task strategy is proposed to comprehensively consider the information from multiple views and evaluate CT image quality as comprehensively as possible. Additionally, each aspect is assigned a corresponding score, which is then integrated to provide an image quality score for the series of CT images obtained from each patient. The entire evaluation process is end-to-end and automated, aiming to minimize the workload of radiologists and improve screening efficiency.

#### Inspiration evaluation

**Fig. 6:**
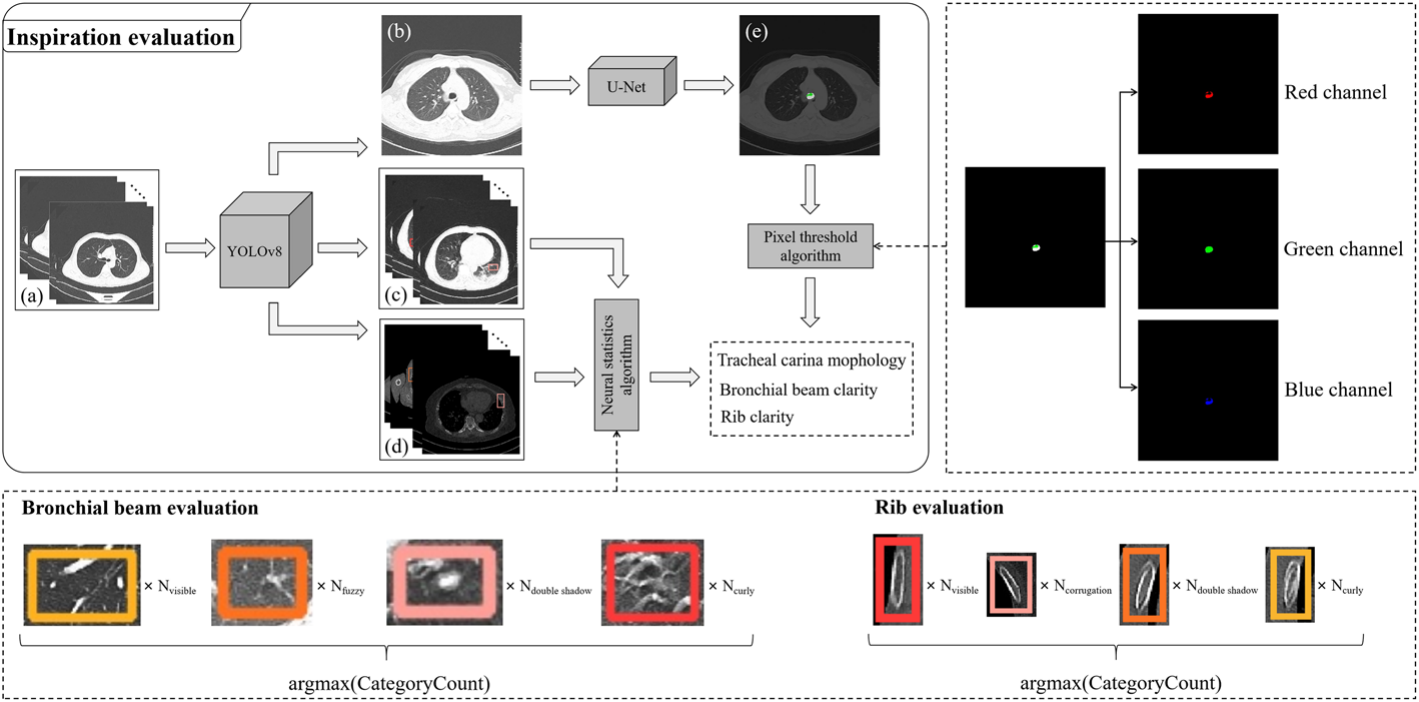
The architecture of the inspiration evaluation. **a** Represents the original CT image sequence, **b** shows the CT image with the tracheal carina visible, **c** displays the CT image sequence with bronchial beam detection results, **d** depicts the CT image sequence with rib detection results, and **e** showcases the segmentation result of the tracheal carina. The dashed rectangular boxes on the right and bottom represents the implementation details of the pixel threshold algorithm and neural statistics algorithm, respectively

To comprehensively evaluate the adequacy of patient inspiration, three aspects (i.e., tracheal carina morphology, bronchial beam clarity, and rib clarity) are considered. the object detection model (YOLOv8) is utilized to obtain ROIs (i.e., tracheal carina, bronchial beam, and rib) from a series of CT images. Since CT scanning saves the results of the current scanning position at regular intervals, typically only one CT image in the series contains the most suitable tracheal carina image for evaluation. For tracheal carina evaluation, further segmentation using the semantic segmentation model (U-Net) is performed based on the YOLOv8 detection result to segment the tracheal carina. The final score is derived from the segmentation result. The architecture and implementation of the three sub-parts of the evaluation are illustrated in Fig. 6.

It is worth noting that due to the similarity between convex and flat tracheal carina (as shown in Fig. 4b, f), a PT algorithm for tracheal carina segmentation result is proposed to address segmentation result misjudgment caused by similar morphology. Inspired by [36], the optimization algorithm improves the segmentation result assessment. Additionally, many CT images in a series of CT images contain detection results for bronchial beams and ribs. Thus, a NS algorithm, aiming to mimic the assessment paradigm of radiologists, is proposed. These two optimization algorithms will be described in the next section.

*Optimization algorithms* In this study, the images used are three-channel images, i.e., the red, green, and blue channels. The segmentation results of the tracheal carina can be classified based on the following rules: a white mask represents a convex morphology, a green mask represents a flat morphology, and a red mask represents a concave morphology. The PT algorithm involves computing the three-channel values of each pixel in the image and then classifying each pixel based on the set threshold values. The calculation process of the PT algorithm is illustrated in the dashed rectangular box on the right side of Fig. 6.

For a pixel, if the values of the red, green, and blue channels simultaneously fall within the range of [200, 255], the count of white pixels is incremented by one. If the value of the red channel falls within the range of [150, 255], the count of red pixels is incremented by one. Similarly, if the value of the green channel falls within the range of [150, 255], the count of green pixels is incremented by one. The threshold-based decision formula can be defined as follows:

1 $$\begin{aligned} Pixel\left( R(x,y),G(x,y),B(x,y)\right) =\left\{ \begin{aligned} &White, \quad 200\leqslant \left\{ R(x,y),G(x,y),B(x,y)\right\} \leqslant 255, \\ &Green, \quad 150\leqslant G(x,y) \leqslant 255, \\ &Red, \qquad 150\leqslant R(x,y) \leqslant 255, \end{aligned}\right. \end{aligned}$$

where *Pixel* represents the current pixel, and *R*(*x*, *y*), *G*(*x*, *y*), *B*(*x*, *y*) represents the values of the red, green, and blue channels of the current pixel, respectively.

Then, the above formula is applied to each pixel in the image, and accumulates the number of white pixels, red pixels, and green pixels separately. The morphology represented by the highest count among these three categories will determine the current morphology of the tracheal carina. The formula is as follows:

2 $$\begin{aligned} Morphology=\left\{ \begin{aligned} &Convex, argmax(C(white),C(green),C(red))=C(white), \\& Flat, argmax(C(white),C(green),C(red))=C(green), \\ &Concave, argmax(C(white),C(green),C(red))=C(red), \end{aligned}\right. \end{aligned}$$

where *argmax*() is a function that returns the parameter with the highest count among the given parameters. *C*() represents the counting function, which calculates the number of pixels corresponding to the given color parameters.

Since radiologists typically do not rely solely on the morphology of bronchial beams and ribs in a single CT image to get the final result but rather review multiple images repeatedly, this study uses an object detection model (YOLOv8) to mimic the radiologist’s process of searching for ROIs. The model can detect ROIs in multiple images. However, due to the limitations of model accuracy, not all detection results are correct. Therefore, a NS algorithm aims to mimic the cognitive and memory abilities of the human nervous system. In this study, it emulates the process where radiologists find ROIs and store cognitive information in the “memory storage system” of the brain. After reviewing all the images, the final assessment is made based on this information. The calculation process of the NS algorithm is shown in the dashed rectangular box at the bottom of Fig. 6.

In this study, the clarity of bronchial beams is classified into four categories: visible, fuzzy, double shadow, and curly. Similarly, the clarity of ribs is also classified into four categories: visible, corrugation, double shadow, and multiple shadows. For a series of CT images of a patient, the NS algorithm counts the number of detection boxes for each category and determines the category with the highest count as the final category assessment. The computation formula for the algorithm is as follows:

3 $$\begin{aligned} FinalCategory=argmax\left( CategoryCount\right) , \end{aligned}$$

where *argmax*() denotes the function that returns the category with the maximum count, and *CategoryCount* represents the count of detection boxes for each category.

The classification rules mentioned above will be described in detail in Classification rules and scoring criteria.

#### Position evaluation

The position evaluation aims to evaluate whether the patient has a proper position based on three aspects: the position of the body within the scanning FOV, accurate alignment of the scan baseline at the beginning and end, and whether the arms are raised above the head. Prior to performing a CT scan, it is necessary to determine the start and end positions of the scan in order to define the scanning range. This helps to avoid unnecessary radiation exposure to additional body areas. By precisely defining the scan range, only the relevant ROI is exposed to radiation, minimizing radiation dose to other parts of the body that are not required for diagnostic purposes. This targeted approach helps in optimizing the scan parameters and reducing potential risks associated with excessive radiation exposure. It is important to ensure that the body does not deviate from the scanning field of view as it can lead to the occurrence of artifacts. When the body is positioned outside the intended scanning area, it may result in incomplete imaging of certain structures, causing truncation artifacts in the final image. Therefore, maintaining proper alignment and positioning of the body within the scanning field of view is crucial to obtain high-quality images and minimize the occurrence of artifacts. In addition, another factor that can contribute to the occurrence of artifacts is when the arms are not raised above the head. During a CT scan, if the arms are positioned incorrectly, such as being placed at the sides of the body, they may cause shadows or streaks in the final image, resulting in artifacts. Therefore, it is important to ensure that the patient’s arms are positioned correctly and raised above the head to minimize the occurrence of such artifacts and ensure image quality.

**Fig. 7:**
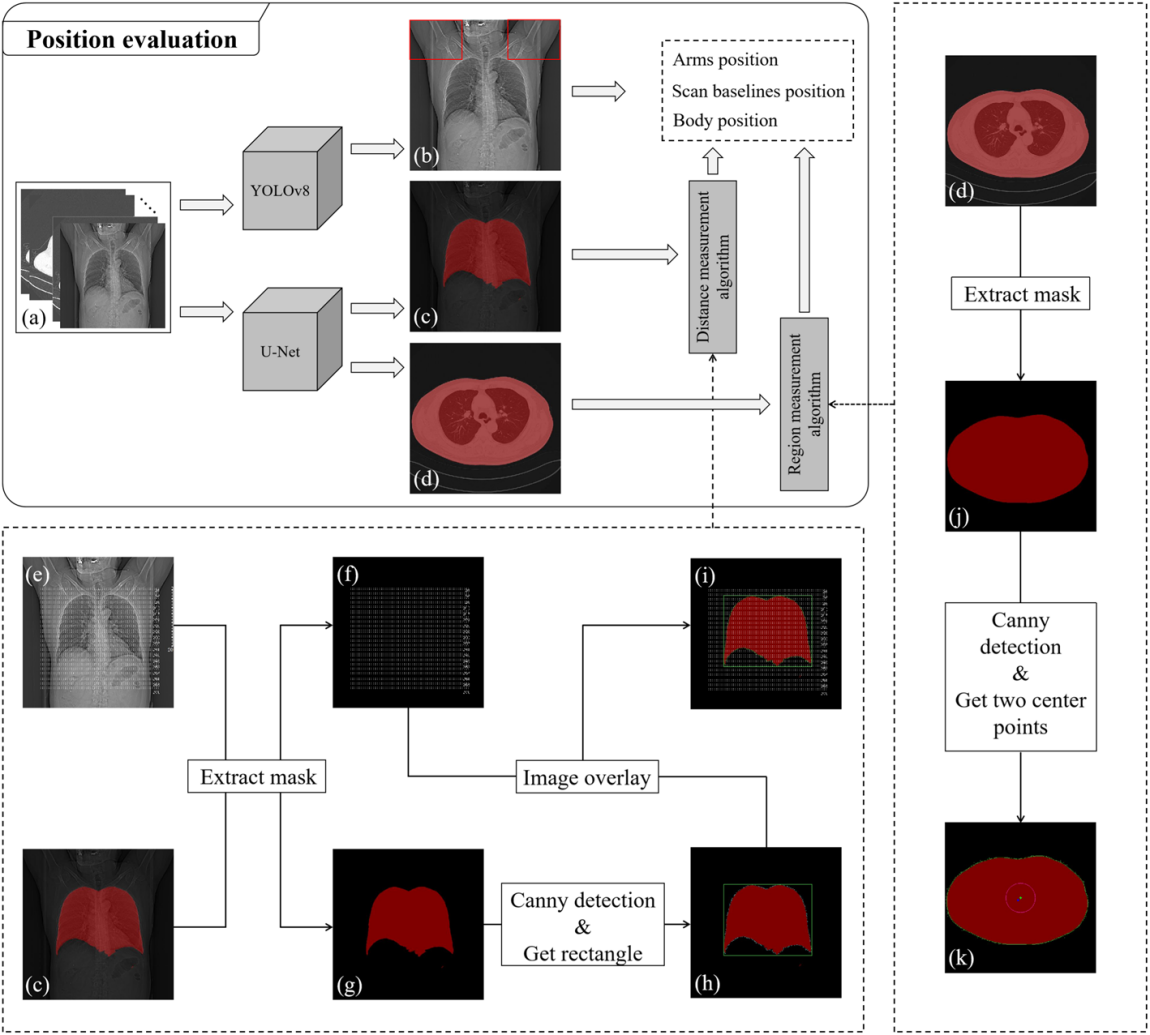
The flowchart of three sub-evaluations in the position evaluation. **a** Is the original CT image sequence, **b** shows the detection result for the arms position evaluation from **a** after YOLOv8 detection. **c**, **d** depict the lung contour segmentation result used for the scan baseline position evaluation and the body contour segmentation result used for the body position evaluation from **a** through U-Net segmentation, respectively. The dashed rectangle box on the bottom illustrates the implementation details of the region measurement algorithm. **e** Is the CT image with the scan baseline, **f** is the extracted scan baseline image from **e**, **g** is the lung contour mask extracted from **c**, **h** is the result after canny detection of the lung contour mask in **g**, with the detected result surrounded by a green rectangle, and **i** is the overlay result of **f** and **h**, which is the final algorithm result. The dashed rectangle box on the right represents the implementation details of the distance measurement algorithm. **j** Is the body contour mask extracted from **d**, **k** is the result after canny detection of the body contour mask in **j**, where blue points indicate the center points of the body contour, and green points represent the center of a circle with a radius of 50 pixels

The object detection model (YOLOv8) and the semantic segmentation model (U-Net) are also used to obtain ROIs (body contour, lung contour, and arms) from a series of CT images. While U-Net provides segmentation results for the body and lung contours, this alone is insufficient to determine whether the body is positioned at the center of the scanning FOV or to assess the alignment accuracy of the start and end scan baselines. Therefore, the region measurement algorithm is proposed to determine the position of the body within the scanning FOV, and the distance measurement algorithm is proposed to assess the alignment accuracy of the start and end scan baselines. The architecture design and implementation of the three sub-parts of the assessment are illustrated in Fig. 7. The two decision algorithms will be described in the next section.

*Decision algorithms* The region measurement algorithm aims to further determine whether the patient’s body is centered within the scanning FOV using the body contour segmentation result obtained from the semantic segmentation model (U-Net). Specifically, after obtaining the body contour segmentation image, the algorithm calculates the center point of the image and draws a circular region with a radius of 50 pixels around that center point. Additionally, the algorithm obtains the center point of the body contour. The dashed box on the right side of Fig. 7 presents the results of body contour segmentation before and after undergoing the region measurement algorithm, where the center of the circle is represented by a green dot and the center point of the body contour is represented by a blue dot. The details of the algorithm are elaborated below.

To obtain a better body contour, the first step is to convert the image to grayscale. This conversion removes the color information from the image and represents it in shades of gray. This simplifies the image and reduces the influence of color variations on the subsequent processing steps. After converting the image to grayscale, the next step is to perform binarization. Binarization is the process of converting the grayscale image into a binary image, where each pixel is classified as either black or white based on a certain threshold value. Pixels with intensity values below the threshold are set to black, representing the background, while pixels with intensity values above the threshold are set to white, representing the foreground (body). For the region measurement algorithm, the threshold value is set to 5 and can be formulated as:

4 $$\begin{aligned} B_{r}\left( x,y\right) =\left\{ \begin{matrix} 1, &{} I(x,y)\geqslant 5, \\ {} &{} \\ 0, &{}I(x,y)< 5, \end{matrix}\right. \end{aligned}$$

where *I*(*x*, *y*) represents a grayscale image, and (*x*, *y*) represents the pixel coordinates in the image. If the grayscale value of a pixel is less than the threshold value of 5, the pixel is classified as black. If the grayscale value of a pixel is greater than or equal to the threshold value of 5, the pixel is classified as white. $B_r(x, y)$ represents the resulting binary image after thresholding, where its values are either 0 or 1, corresponding to black and white, respectively. By applying grayscale conversion followed by binarization, we can obtain a binary image where the body contour appears as a distinct white region against a black background. This binary image can then be used for further processing and analysis, such as extracting the body contour and performing measurements.

To determine if a blue point falls within a circle with the green point as its center, the following formulas can be used:

5 $$Distance=\sqrt{(x_{blue} - x_{green})^2 + (y_{blue} - y_{green})^2}$$

6 $$\begin{aligned} Decision_{region}= \left\{ \begin{aligned}& Inside, distance< radius, \\ &Outside, distance\geqslant radius, \end{aligned}\right. \end{aligned}$$

where ($x_{blue}$, $y_{blue}$) represents the coordinates of the blue point, ($x_{green}$, $y_{green}$) represents the coordinates of the green point center of the circle), and the radius represents the desired radius of the circle, which is set of 50 in the experiment through empirical study. The distance between the blue and green points is calculated using the Euclidean distance formula. If the distance is less than or equal to the radius, the blue point is considered to be inside the circle.

The distance measurement algorithm aims to further assess the alignment accuracy of the start and end scan baselines by utilizing both the lung contour segmentation results from the semantic segmentation model (U-Net) and the overlayer from the DICOM file. Specifically, after obtaining the lung contour segmentation image, it is tightly enclosed within a rectangular box, which is then overlaid with the overlayer image. The dashed box at the bottom of Fig. 7 displays the results of lung contour segmentation before and after undergoing the distance measurement algorithm. The details of the algorithm are elaborated below.

In order to obtain better lung contours, the aforementioned process was applied to the lung contour segmentation image as well. This involves converting the original image to grayscale and then obtaining the binary image. It is worth noting that the threshold for binarization is also set to 5, but in this case, the resulting binary image is represented as $B_d(x, y)$.

To assess the alignment accuracy of the start and end scan baselines, it is necessary to measure the distance between the upper edge of the rectangular box and the starting scanning baseline, as well as the distance between the lower edge of the rectangular box and the ending scanning baseline, then the followings formula can be used:

7 $$Distance_{start}=y_{boxtop} - y_{startline}$$

8 $$Distance_{end}=y_{endline} - y_{boxbottom},$$

where $y_{boxtop}$ and $y_{boxbottom}$ represent the y-coordinates of the top and bottom edges of the rectangular box, respectively. $y_{startline}$ and $y_{endline}$ represent the y-coordinates of the starting and ending scanning baselines, respectively. The decision process can be expressed by the following formulas:

9 $$\begin{aligned} Decision_{distance}=\left\{ \begin{aligned}& Accurate, \qquad D\leqslant T,\\ &Inaccurate, \quad D> T, \end{aligned}\right. \end{aligned}$$

where *D* can be either $distance_{start}$ or $distance_{end}$, depending on the baseline being evaluated. *T* represents the threshold for decision-making, which is set to 15 in this experiment through empirical study.

#### Radiation protection evaluation

**Fig. 8:**
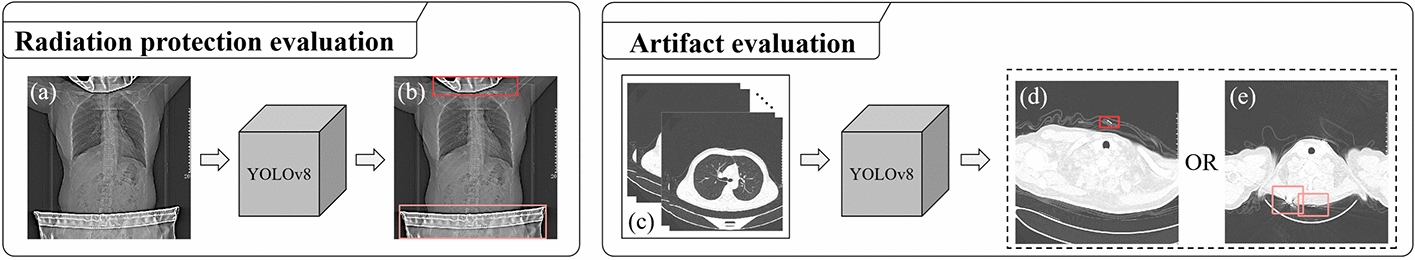
The architectures of radiation protection and artifact evaluations. In the radiation protection evaluation, **a** is the original CT image, and **b** shows the result of **a** after YOLOv8 detection, with protective products on the neck and abdomen surrounded by red and pink boxes, respectively. For artifact evaluation, **c** is the original CT image sequence, either **d** or **e** displays the result of **c** after YOLOv8 detection, with detected foreign matter surrounded by red boxes in **d**, and radial artifacts surrounded by pink boxes in **e**

Radiation protection evaluation aims to determine whether patients have undergone proper radiation protection based on the wearing of radiation-protective products on their neck and abdomen. Since different parts of the body have varying levels of sensitivity to radiation, it is common practice to have patients wear lead-based radiation-protective products on radiation-sensitive areas such as the thyroid gland and reproductive organs before undergoing a CT scan. Figure 1a shows an example of a patient wearing radiation-protective products on both the neck and abdomen. In this assessment, the object detection model (YOLOv8) is utilized to detect the presence of radiation-protective products. The implementation process for the evaluation in this section is illustrated in the rounded rectangle at the left of Fig. 8.

#### Artifact evaluation

Artifact evaluation aims to evaluate whether patients have removed metal jewelry or other objects that could interfere with the quality of the CT scan images prior to the examination. Figure 1f demonstrates a metal artifact caused by a metallic object. In this assessment, the object detection model (YOLOv8) is employed to detect metal artifacts and other foreign objects. The implementation process for the evaluation in this section is illustrated in the rounded rectangle at the right of Fig. 8.

#### Multi-view fusion

In this section, the multi-view fusion strategy will be elaborated in detail. This study employs a multi-view fusion analysis and evaluation approach utilizing information from three scan planes (coronal, axial, and sagittal). Different evaluation tasks utilize different scan planes. For instance, the axial and sagittal planes are predominantly used for inspiration evaluation, the axial and coronal planes for position evaluation, the coronal plane for radiation protection evaluation, and the axial plane for artifact evaluation. Therefore, the multi-view fusion strategy is primarily built upon a multi-task evaluation foundation.

Anatomically speaking, the coronal plane divides the human body into front and back portions along its long axis, while the sagittal plane divides the body into left and right portions. The axial plane, on the other hand, divides the body into upper and lower sections from a front-to-back perspective. As organs exhibit distinct features across these three scan planes, a deep learning model extracts task-specific medical image features from these planes to enhance task specificity and robustness.

By leveraging the characteristics of the specific medical images from the three scan planes, the deep learning model can better tailor its performance to each evaluation task, thus enhancing its capability to achieve accurate and robust evaluations.

#### Experimental settings

*Classification rules and scoring criteria* For a better qualitative and quantitative analysis of the experimental results, the classification rules and scoring criteria presented in Table 8 were utilized to categorize and score the outcomes of each evaluation sub-part. Subsequently, for the inspiration evaluation task, a score not exceeding 20 points (including 20 points) was defined as “inacceptable” image quality, while a score greater than 20 points was considered “acceptable” image quality. Additionally, a similar binary classification was applied to the overall evaluation task: a score not exceeding 40 points (including 40 points) was designated as “inacceptable” image quality, while a score greater than 40 points was labeled as “acceptable” image quality.

The rationale behind these definitions is as follows: the maximum achievable score for the inhalation assessment task is 40 points, and for the overall evaluation task, it is 80 points. In this study, four experienced CT image quality diagnostic physicians were invited to perform blind reading, evaluating the resolution of lesions and major structures in CT images. The image clarity required for clinical diagnosis, where diagnostic reports could be issued, was set as the “acceptable” standard. Hence, image quality scores needed to exceed at least 50% of the total available score for an image to be considered “acceptable”.

**Table 8:** The classification rule and scoring criteria

Artifact		Scan baseline	
Classification	Score	Classification	Score
No exist	10	Beginning and end baselines are correct position	10
Foreign matter	5	Beginning or end baseline is correct position	5
Radial artifact	0	Beginning and end baselines are incorrect position	0

Tracheal carina		Radiation protection	
Classification	Score	Classification	Score
Convex	10	Head and abdomen all radioprotected	10
Flat	5	Only head or abdomen radioprotected	5
Concave	0	No exit	0

Rib		Bronchial beam	
Classification	Score	Classification	Score
Visible	15	Visible	15
Corrugation	10	Fuzzy	10
Double shadow	5	Double shadow	5
Multiple shadows	0	Curly	0

Arms position		Body position	
Classification	Score	Classification	Score
Arms are raised over head	5	The body is entered in the scan FOV	5
Arms are not raised over head	0	The body is not entered in the scan FOV	0

*Implementation details* The experiments in this study include ablation, comparison, and observer study. For the proposed M$^2$IQA method, YOLOv8 is used as the object detection model, and U-Net is used as the semantic segmentation model. All model training adopts transfer learning strategies to better extract image features. YOLOv8 utilizes pre-trained weights on the COCO (Common Objects in Context) [37] dataset, while U-Net utilizes pre-trained weights on the ImageNet [38] dataset. The images are standardized by subtracting the mean and dividing by the standard deviation of the image. The parameter settings for the models are shown in Table 9.

**Table 9:** The parameter and hyperparameter settings of the model

Model	Image size	Optimizer	Initial learning rate	Loss function
YOLOv8	640 x 640	Stochastic gradient descent (SGD)	0.01	Binary cross entropy
U-Net	512 x 512	Adaptive moment estimation (ADAM)	0.0001	Cross entropy

Data augmentation techniques were employed to address the issue of limited data. Different augmentation methods were applied specifically to different evaluation parts, aiming to obtain more informative training data and improve the model’s robustness. Detailed descriptions of the data augmentation methods are provided in Table 10. The early stopping strategy was also utilized during model training. The model’s performance was evaluated on the validation dataset every 10 epochs, and training would be stopped and the best-performing model would be saved when optimal performance was achieved. Subsequently, the saved model was tested on the test dataset. All experiments were conducted on a workstation equipped with two NVIDIA RTX 2080Ti GPUs. Python was used as the programming language, and deep learning frameworks such as PyTorch and TensorFlow were employed.

**Table 10:** Data augmentation methods

	Inspiration	Position	Radiation protection	Artifact
Vertical flipping	$\checkmark$	$\checkmark$	$\checkmark$	$\checkmark$
Horizontal flipping	$\checkmark$	$\checkmark$	$\checkmark$	$\checkmark$
Random scaling	$\checkmark$	$\checkmark$	$\checkmark$	
Random cropping and padding	$\checkmark$	$\checkmark$	$\checkmark$	
Gaussian noise	$\checkmark$			
Gaussian blur	$\checkmark$			
Contrast limited adaptive histogram equalization	$\checkmark$		$\checkmark$	$\checkmark$
Edge-enhancing	$\checkmark$			$\checkmark$
Brightness randomization	$\checkmark$		$\checkmark$	$\checkmark$
Perspective transformation	$\checkmark$	$\checkmark$		
Motion blur	$\checkmark$			
Additional images	2680	1234	690	2090

Evaluation metrics In our experiments, sensitivity, precision, specificity, and F1-score are used as evaluation metrics to assess the performance of the model. The definitions of these metrics are as follows:

10 $$\begin{aligned} \begin{aligned} Sensitivity&=\frac{\text{TP}}{\text{TP}+\text{FN}},\\ Precision&=\frac{\text{TP}}{\text{TP}+\text{FP}},\\ Specificity&=\frac{\text{TN}}{\text{FP}+\text{TN}},\\ F1\text{-}score&=\frac{2*precision*sensitivity}{precision+sensitivity}, \end{aligned} \end{aligned}$$

where TP, TN, FP, and FN represent the counts of true positive, true negative, false positive, and false negative samples, respectively. These metrics are calculated for each individual subclass within the overall classification. The average values across all subclasses are then taken as the final results. Specifically, for each evaluation category (e.g., tracheal carina in the inspiration evaluation task), the metric values are recorded for each subclass (convex, flat, concave) in the test dataset. At the end of the testing process, the average values across the three classes are computed. In our experiments, precision, sensitivity, and F1-score are considered the most important evaluation criteria for validating the correctness and effectiveness of the proposed learning framework.

## Data Availability

All the data used and/or analyzed during the current study are available from the corresponding author upon reasonable request.
